# One Health horsepower in hemostasis: SEL1Lecting stable platelet adhesion

**DOI:** 10.1172/JCI202267

**Published:** 2026-02-16

**Authors:** Caitlin D. Schneider, James P. Luyendyk

**Affiliations:** 1 Department of Pharmacology and Toxicology, and; 2 Department of Pathobiology and Diagnostic Investigation, College of Veterinary Medicine, Michigan State University, East Lansing, Michigan, USA.

## Abstract

Comparative biology approaches have produced foundational discoveries in the mechanisms underlying thrombosis and hemostasis. In this issue of the *JCI*, work by Dahlgren and colleagues continues in this tradition using an approach that integrated a multispecies investigation of conserved function with genomic exploration and discovery. Dahlgren et al. describe the identification of pathogenic variants in the ER-associated degradation pathway protein SEL1L in a rare platelet disorder affecting horses. After establishing a conserved role for SEL1L in zebrafish, mouse, and human platelet function, the study found evidence for SEL1L variants in association with bleeding phenotypes in human GWAS. Altogether, the findings elucidate a previously unrecognized component of platelet function, laying the groundwork for mechanistic explanation of a subset of human bleeding phenotypes and providing a powerful endorsement of integrative, collaborative research.

Major advances have been made in defining the precise origin of bleeding disorders. Among many changes, the fundamental basis can now include a reduction in key hemostatic proteins (e.g., von Willebrand factor [VWF], procoagulants) or specific defects in platelet function. However, a substantial challenge remains in identifying the molecular mechanisms underlying bleeding disorders. For most patients (up to 70%) referred for bleeding tendency, a formal diagnosis includes “unknown cause” (1). Considerable scientific and clinical energy is being invested in uncovering these mechanisms (2), and right out of the starting gate, it may be useful to change our tack to an integrated approach. The One Health approach involves collaboration between human, animal, and environmental health specialists to address health outcomes across disciplines in a manner that could not be achieved by a single-pronged approach. Under this paradigm, the lines demarcating human, animal, and environmental health disciplines become incredibly porous as interconnections at multiple levels are recognized. One Health approaches are becoming more frequently implemented in issues such as antimicrobial resistance or zoonotic disease.

In this issue of JCI, Dahlgren et al. have loosened the reins on disciplinary boundaries and describe impactful observations driven by a collaboration that seems serendipitous (3). Such is often the case when game-changing discoveries result from viewing challenges through a One Health lens. Described as a multispecies investigation by the research team, the story told by Dahlgren et al. transcends the boilerplate approach of many multispecies investigations by tracing the conserved functions of a novel candidate protein from an equine disease to unexplained phenotypes in human GWAS. This study represents truly unbridled thinking that emphasizes acceleration of the current path, or even a paradigm shift, in strategies to identify the molecular basis of bleeding disorders. Embracing the interconnectedness of species, as done by Dahlgren et al., unlocks a new opportunity to understand disease mechanisms and improve outcomes in both human and veterinary medicine. By investigating a unique bleeding disorder in horses, extending their observations to experimental settings in multiple species, and hitching the results to genomic evidence and human platelets, the authors demonstrate how cross-species collaboration can reveal conserved pathways in platelet biology (Figure 1).

## SEL1L in platelets: off the beaten path

Dahlgren et al. sought to uncover the genetic basis of atypical equine thrombasthenia (AET) (3), a rare inherited platelet disorder in thoroughbred horses (4). Levels of the coagulation protease thrombin and its proteolytic target fibrinogen were normal in AET-affected horses. Yet, functionally, platelets from horses with AET displayed a unique agonist-specific defect in activation, namely, an impaired response to thrombin stimulation, a robust activator of platelets. Through whole-genome sequencing and association studies, the authors identified a missense variant in SEL1L (c.1810A>G p.Ile604Val) as the likely causative mutation. SEL1L is a key adaptor protein in the ER-associated degradation (ERAD) pathway (5), which functions as a quality control manager for proteins in cells and operates by targeting misfolded proteins for degradation.

Although the role of SEL1L has been vetted in nucleated cells, its function in platelets remained unclear. Dahlgren et al. set out to identify a role for SEL1L in anucleate platelets (3). Expression of the AET-associated variant decreased SEL1L protein expression and impaired platelet spreading on collagen in models based on equine tissue. Importantly, the team then extended their investigation beyond the paddock using human megakaryocytes, conditional knockout mice, and zebrafish models. Across these models, SEL1L deficiency consistently resulted in defective platelet (or thrombocyte) adhesion to sites of endothelial injury, highlighting a conserved role for SEL1L in hemostasis. GWAS in humans linked SEL1L variants to bleeding phenotypes, suggesting relevance in both human and veterinary medicine. Collectively, these findings position SEL1L as a key player in platelet biology and underscore the power of One Health research as an approach to potentially move the needle on identifying new bleeding disorders in animals and humans.

SEL1L seems to be connected to other aspects of hemostasis beyond its role in platelet activation. Prior studies demonstrated that the SEL1L-HRD1 ERAD complex regulates the production of coagulation proteins by liver parenchymal cells (hepatocytes) (6). SEL1L-deficient hepatocytes retained fibrinogen, leading to its intracellular accumulation and the formation of fibrinogen-rich inclusion bodies (6). Similar fibrinogen-containing hepatic inclusion bodies have been reported clinically in individuals with hepatic fibrinogen storage disease (HFSD). In both congenital (mutations in fibrinogen Aα and γ chains) and acquired (e.g., due to hepatocellular carcinoma or hepatitis B virus infection) HFSD, distinct protein profiles within these hepatic inclusion bodies suggest impaired autophagy and lysosome-mediated degradation, with pharmacological stimulation of autophagy in HFSD holding therapeutic promise (7). Taken together, these findings suggest a relationship between SEL1L-HRD1 ERAD complexes and diseases of hepatic fibrinogen accumulation, with the potential for there to be multiple pressure points linking SEL1L to hemostasis.

## One Health paradigms have powered major antithrombotic advances

The field of hemostasis and thrombosis is full of examples where comparative medicine and integrative biology have bridged the gap between species, leading to breakthroughs that benefit both veterinary and human medicine (8). Classic studies of canine hemophilia, for instance, provided foundational models for understanding factor VIII and IX deficiencies, ultimately informing the development of replacement therapies for humans (9). Similarly, research into bovine and murine models of von Willebrand disease (VWD) has illuminated the molecular underpinnings of platelet adhesion and aggregation. The list goes on. Diagnosis of VWD in horses is directly enabled by tests that were developed for human medicine. The key regulator of VWF, a protease termed ADAMTS13, varies tremendously in sequence and structure across species (10). Defining this cross-species variation has allowed reciprocal development of improved assays based on bovine proteins that have utility in humans and beyond (11).

The “mane” event, however, is the history of major life-saving antithrombotic drug pharmacology delivered by One Health partnerships. The history of warfarin development begins with a production agriculture twist, unanticipated deaths in cattle, and a genuine veterinary toxicologist origin story worthy of a true-crime documentary (12). The story of aspirin is much the same, with tremendous pharmacology built over centuries on principles that the bark of a willow tree has medicinal properties (13). Anticoagulant heparin is routinely isolated from the intestines of pigs, leaving the supply of a life-saving anticoagulant drug subject to global supply chains, antimicrobial resistance and regulation, and disease detection by local, state, and federal veterinary diagnostic laboratories (14). For those who care for patients with heparin-induced thrombocytopenia (HIT), thank your favorite Michigan lake and the hematophagous organisms that call it home, as they produce hirudin, from which the knowledge originated to produce bivalirudin, a common medicine administered to patients with HIT (15).

With origin stories like these, it may not be putting the cart before the horse to suggest that the study by Dahlgren et al. (3) should spur action in studies seeking the next target and/or pathway regulating hemostasis and thrombosis. Indeed, there are needs in veterinary medicine that are awaiting collaborative approaches, including thrombotic complications of infectious disease in horses, such as equine herpesvirus myeloencephalopathy (16, 17), the impact of which is emphasized by an outbreak noted just prior to the writing of this commentary. Experts in thrombosis are encouraged to gallop into action in this area.

## Betting on cross-disciplinary and cross-species exploration

**Figure 1:**
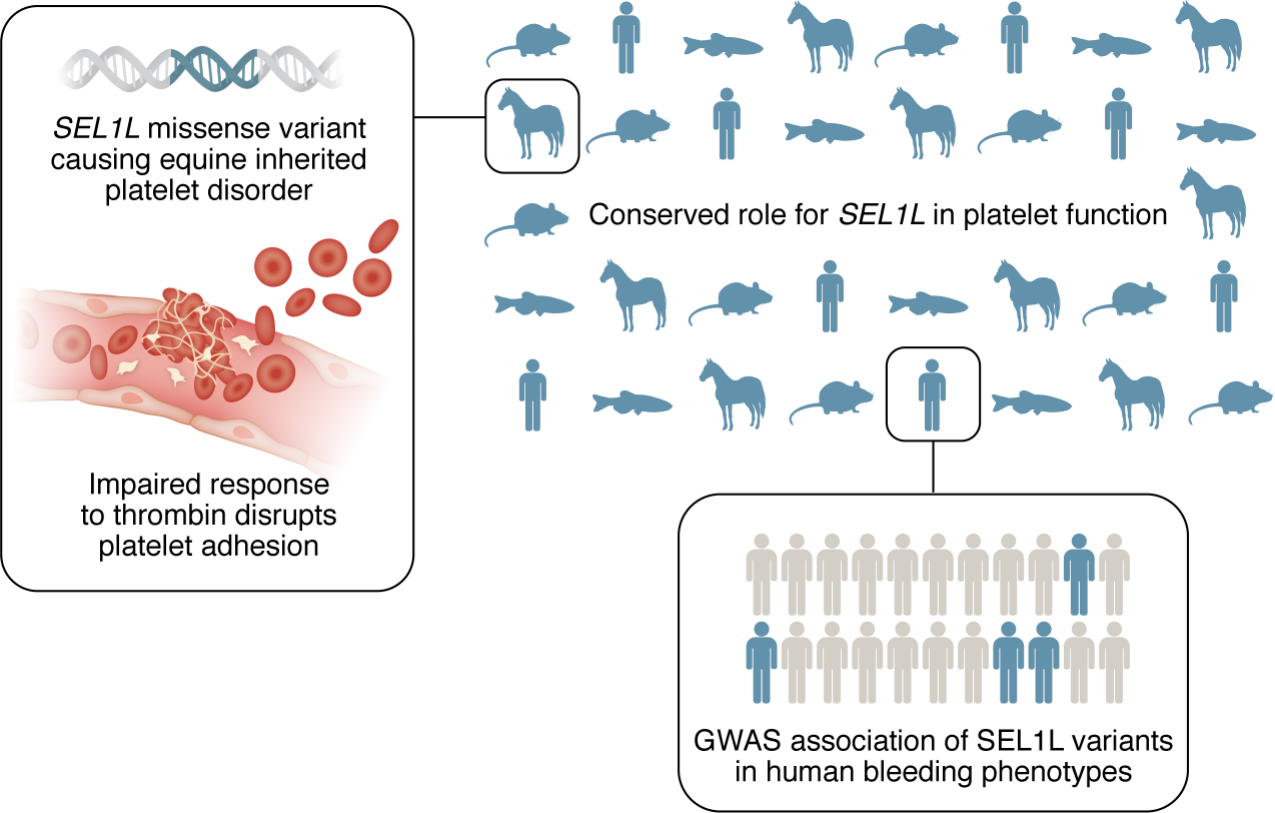
Multispecies research to advance understanding of bleeding disorders. Dahlgren et al. (3) used a multispecies One Heath–framed approach to uncover mechanisms underlying AET, a rare bleeding disorder in horses. After identifying missense mutations in SEL1L in the equine disease, mechanistic implications were determined using mice, zebrafish, and human platelets, and the translational implications include identification of variants of SEL1L potentially connected to bleeding disorders of unknown origin in humans. There is abundant potential for this approach, pairing challenges in veterinary and human medicine, to address smoldering challenges in the field of hemostasis and thrombosis.

The late Robert Redford’s character in the 1998 movie *The Horse Whisperer* stated, “Truth is, I help horses with people problems.” The work of Dahlgren et al. calls this quote into action and identifies reciprocity in its central theme (3). Specifically, individuals bringing the capacity to bridge research expertise in human and veterinary medicine can provide strong mechanistic evidence of the basis of a rare disease in horses (Figure 1). Indeed, Dahlgren et al. have uncovered what we believe to be a novel origin of unknown bleeding not only in horses, but also in humans, informed by expertise in hematology and fully customizable genetic approaches in zebrafish. Whether from funding agencies or philanthropy, now is the time to pony up support for partnerships like those typified by Dahlgren et al.’s study. One Health and cross-species critical thinking can address the grand challenges at the intersection of human, animal, and environmental health.

## Funding support

This work is the result of NIH funding, in whole or in part, and is subject to the NIH Public Access Policy. Through acceptance of this federal funding, the NIH has been given a right to make the work publicly available in PubMed Central.

- NIH grant R01 DK136733 to JPL.
- The Albert C. and Lois E. Dehn Endowment to Michigan State University for Veterinary Medicine (Pathobiology and Diagnostic Investigation) to JPL.

## Version 1 02/16/2026

Electronic publication
